# Genomic characterization revealing the high rate of *tet*(X4)-positive *Escherichia coli* in animals associated with successful genetic elements

**DOI:** 10.3389/fmicb.2024.1423352

**Published:** 2024-06-24

**Authors:** Li Shao, Changbu Wu, Chengjuan Li, Ruowen He, Guanping Chen, Dandan Sun, Yanxian Yang, Yu Feng, Guili Zhang, Bin Yan, Min Dai, Guo-Bao Tian, Lan-Lan Zhong

**Affiliations:** ^1^School of Medicine, Xizang Minzu University, Xianyang, Shanxi, China; ^2^Advanced Medical Technology Center, The First Affiliated Hospital, Zhongshan School of Medicine, Sun Yat-sen University, Guangzhou, China; ^3^State Key Laboratory of Oncology in South China, Sun Yat-sen University Cancer Center, Guangzhou, China; ^4^Program in Pathobiology, The Fifth Affiliated Hospital, Zhongshan School of Medicine, Sun Yat-sen University, Guangzhou, Guangdong, China; ^5^Key Laboratory of Tropical Diseases Control (Sun Yat-sen University), Ministry of Education, Guangzhou, China; ^6^Microbiome Medicine Center, Department of Laboratory Medicine, Zhujiang Hospital, Southern Medical University, Guangzhou, China; ^7^Department of Immunology, School of Medicine, Sun Yat-sen University, Shenzhen, China; ^8^Department of Neonatal Surgery, Guangzhou Women and Children's Medical Center, Guangzhou, China; ^9^School of Laboratory Medicine, Chengdu Medical College, Chengdu, Sichuan, China

**Keywords:** *Escherichia coli*, tigecycline resistance, *tet*(X4), colonization, bioinformatics analyses

## Abstract

**Introduction:**

The rapid spread of plasmid-mediated *tet(X4)* conferring high tigecycline resistance poses a significant threat to public health. *Escherichia coli* as the most common pathogen which carries *tet(X4)* has been widely disseminated in China. Thus, comprehensive investigations are required to understand the mechanism of transmission of *tet(X4)*-positive *E. coli*.

**Methods:**

In this study, a total of 775 nonduplicate samples were collected in Guangdong, China from 2019 to 2020. We screened for *tet(X4)*-positive *E. coli* by PCR amplification and species identification. Furthermore, we analyzed the phylogenetics and genetic context of *tet(X4)*-positive *E. coli* through whole-genome sequencing and long-reads sequencing.

**Results:**

Overall, 146 (18.84%) *tet(X4)*-positive *E. coli* were isolated, comprising 2 isolates from humans and 144 isolates from pigs. The majority of *tet(X4)*-positive E. coli exhibited resistance to multiple antibiotics but all of them were susceptible to amikacin and colistin. Phylogenetic analysis showed that ST877, ST871, and ST195 emerged as the predominant sequence types in *tet(X4)*-positive *E. coli*. Further analysis revealed various genetic environments associated with the horizontal transfer of *tet(X4)*. Notably, a 100-kbp large fragment insertion was discovered downstream of *tet(X4)*, containing a replicon and a 40-kbp gene cluster for the bacterial type IV secretion system.

**Discussion:**

The high colonization rate of *tet(X4)*-positive *E. coli* in animals suggests that colonization as a key factor in its dissemination to humans. Diverse genetic context may contribute to the transfer of *tet(X4)*. Our findings underline the urgent need for controlling the spread of plasmid-mediated tigecycline resistance.

## Introduction

With the overuse of antibiotics, the problem of bacterial resistance has become increasingly serious, followed by the emergence and prevalence of various superbugs, including carbapenem-resistant *Enterobacteriaceae*, carbapenem-resistant *Acinetobacter baumannii*, which pose a huge threat to human health annually and places a huge burden on healthcare systems (Hsu et al., 2017; Tacconelli et al., 2018). Tigecycline, as third generation tetracycline drug, exerts antibacterial effects by binding to the 30S subunit of the ribosome and inhibiting peptide elongation (Olson et al., 2006). It is recognized as a crucial option against multi-drug resistant bacteria, often referred to as the “last resort.”

However, recently, a large number of tetracycline resistance genes have emerged and become popular (Fang et al., 2020). The plasmid-mediated *tet*(X4) gene, which mediates the high level of tigecycline resistance, was discovered in 2019 (He et al., 2019). In addition, *tet*(X4) is prevalent worldwide, primarily in China (Zhang et al., 2022), and has been found in a variety of sample sources, including patients, farms, slaughterhouses, sewage, and migratory birds (Chen et al., 2019; Cui et al., 2022; Dong, 2022). Thus, the spread of the *tet*(X4) is a public health concern that cannot be ignored.

An important reason for the pandemic of resistance genes is that they tend to transfer in individual samples, which is associated with a variety of transfer elements (Partridge et al., 2018). Previous studies have shown that the transfer of *tet*(X4) is related to several transfer elements, including IS*CR2*, IS*26*, and IS*1R* (Liu et al., 2022). The transmission mode of *tet*(X4) requires further investigation. The type IV secretion system (T4SS), which mediates the horizontal transfer of antibiotic resistance and virulence genes (Christie, 2016), has been found in the *tet*(X4)-positive plasmid (Du et al., 2020). However, the insertion of large fragments containing T4SS associated genes into *tet*(X4) plasmids has rarely reported.

In this study, we described the genomic characteristics of *tet*(X4)-positive *E. coli* from animals and humans in China and the potential element insertions related to T4SS which may associated with high-frequency transfer of *tet*(X4). Therefore, it emphasizes that the sustainability surveillance of *tet*(X4) is imperative.

## Materials and methods

### Epidemiological study

We undertook a retrospective observational cross-sectional study to assess the prevalence of *tet*(X4)-positive *E. coli* in animals, farmers, inpatients and healthy volunteers. A total of 775 nonduplicate samples were collected from two farms and one hospital in Guangdong province, China from 2019 to 2020, including 475 nasal swab samples of pigs in two farms, with farm 1 contributing 419 samples and farm 2 contributing 56 samples, 67 fecal swab samples of pigs, 48 skin swab samples of pig farm workers in farm 1, 172 fecal swab samples of inpatients and 13 fecal swab samples of healthy volunteers.

Samples were cultured on brain heart infusion (BHI) plates containing tigecycline (2 mg/L) and incubated overnight at 37°C. Subsequently, the tigecycline-resistant colonies with the typical colonial morphology of Enterobacteriaceae – namely moist-looking colonies which were smooth, translucent with a regular edge were screened for the presence of *tet*(X4) by PCR using the primers *tet*(X4)-F (5’-AGGAACAGGACACGAATTGC-3′) and t*et*(X4)-R (5’-TTACTGGCGGAGCCGTCTA-3′) (Ding et al., 2020). Species identification of *tet*(X4)-positive isolates was conducted by MALDI-TOF MS (BrukerDaltonik GmbH, Bremen, Germany) and 16S rDNA sequencing.

### Antimicrobial susceptibility testing

The minimum inhibitory concentrations (MICs) of tetracycline (TET), gentamicin (GEN), amikacin (AMK), ampicillin (AMP), imipenem (IMP), ceftazidime (CAZ), cefotaxime (CTX), chloramphenicol (CHL), ciprofloxacin (CIP), fosfomycin (FOS), and trimethoprim-sulfamethoxazole (SXT) were determined using the agar dilution method, excepted tigecycline (TGC) and colistin (CT) were determined using the broth dilution method. The results were interpreted in accordance with the Clinical and Laboratory Standards Institute guidelines (CLSI, 2022), as well as the European Committee on Antimicrobial Susceptibility Testing (EUCAST, 2021).

### Whole-genome sequencing and bioinformatics analysis

Genomes of *tet*(X4)-positive *E. coli* were sequenced using Illumina NovaSeq 6000 platform. After completing quality control of raw sequencing reads using fastp v0.20 (Chen et al., 2018), the contigs were generated using SPAdes v3.13.1 (Bankevich et al., 2012). Contigs <500 bp in length were filtered out.^1^ Coding sequences were annotated using Prokka v1.12. Specific genes were detected by comparing contigs to various corresponding database using Abricate v0.8.7,^2^ including Resfinder database for resistance genes, Virulence Factor Database for virulence genes, and Plasmidfinder database for replicon types. MLST (Multilocus Sequence Typing) was identified using mlst v2.23.0.^3^

### Long-reads sequencing and plasmid analysis

To analyze the *tet*(X4)-carrying plasmids, long-reads sequencing was performed using the PacBio platform. Raw data were assembled using Unicycler v0.4.8. The method of gene annotation and identification was the same as that used for the whole genome analysis mentioned above. Insertion sequences were identified using Isfinder.^4^ T4SS genes were identified using oriTfinder.^5^ The comparison of the genetic environments of *tet*(X4) were performed using Easyfig v2.1 and R package “gggenes.”

### Phylogenetic analysis

Pan-genome was constructed using Roary v3.12.0 with the “- e -- mafft” parameter (Page et al., 2015). Core-genome single nucleotide polymorphism were extracted and filtered using snp-sites (Page et al., 2016) and VCFtools (Danecek et al., 2011), respectively. The phylogenetic tree based cgSNP was constructed using RAxML v8.2.10 (Stamatakis, 2014) with GTR + G model and was visualized using iTOL (Letunic and Bork, 2021). The Minimum spanning tree was constructed using PHYLOViZ 2.0 (Nascimento et al., 2017).

## Results

### Molecular epidemiology of *tet*(X4)-positive *Escherichia coli*

Among the 775 multi-source samples, 146 (18.84%) *tet*(X4)-positive *E. coli* were isolated. Out of these, 144 isolates were obtained from pigs originating from farm 1 (106/486, 21.81%) and farm 2 (38/56, 67.86%). Additionally, 2 isolates were derived from skin swabs of farm workers and inpatients, respectively. Specifically, 26.95% (128/475) of *tet*(X4)-positive *E. coli* were obtained from nasal swabs of pigs, followed by 23.88% (16/67) from fecal swabs of pigs. In contrast, *tet*(X4)-positive *E. coli* appeared sporadically in the skin swabs of pig farm workers (1/48, 2.08%) and anal swabs of inpatients (1/172, 0.58%). It was *tet*(X4) negative in fecal swabs of healthy volunteers.

**Figure 1:**
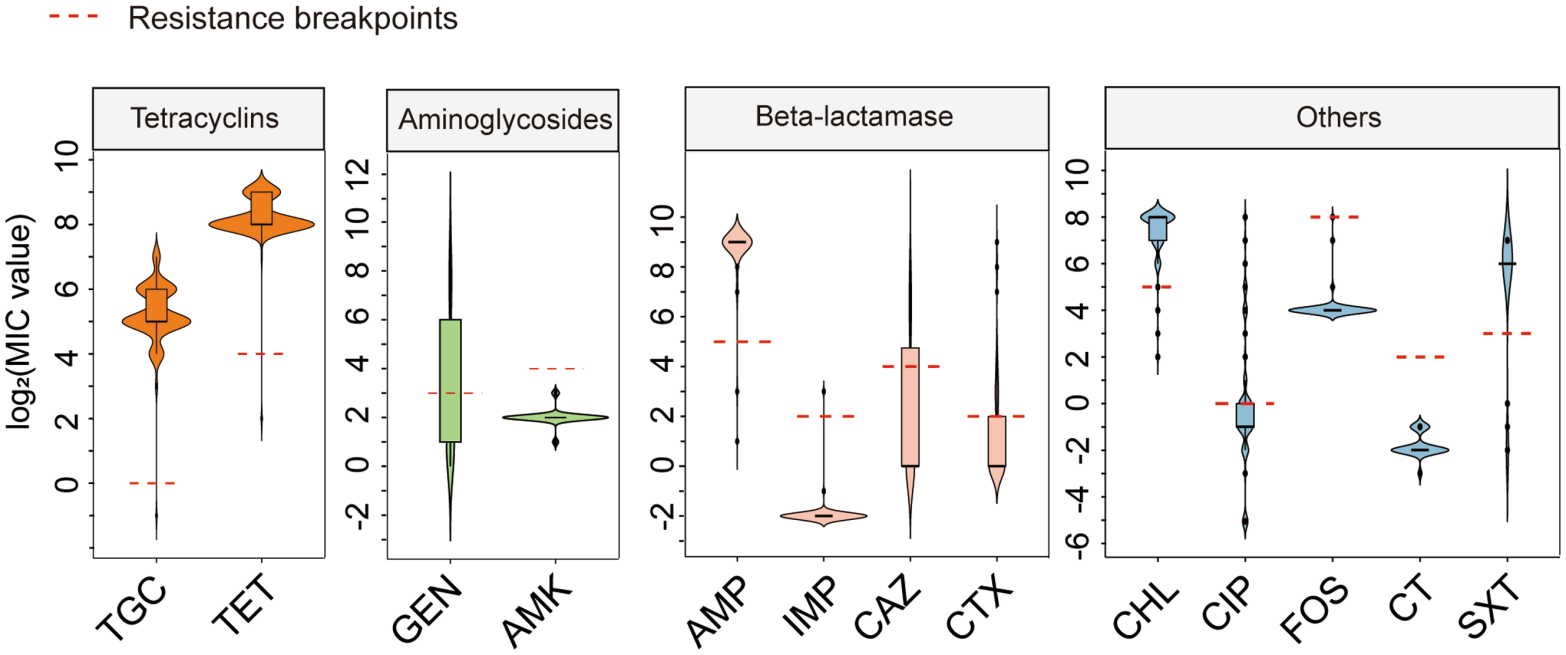
Antimicrobial resistance profiles of 56 *tet*(X4)-positive *E. coli.* TGC, Tigecycline; TET, Tetracycline; GEN, Gentamicin; AMK, Amikacin; AMP, Ampicillin; IMP, Imipenem; CAZ, Ceftazidime; CTX, cefotaxime; CHL, Chloramphenicol; CIP, Ciprofloxacin; FOS, Fosfomycin; CT, Colistin; SXT, Sulfamethoxazole/Trimethoprim. Red dashed line: MIC breakpoint based on CLSI and EUCAST guidelines: TGC > 0.5, TET ≥ 16, GEN ≥ 8, AMK ≥ 16, AMP ≥ 32, IMP≥4, CAZ ≥ 16, CTX ≥ 4, CHL ≥ 32, CIP ≥ 1, FOS ≥ 256, CT ≥ 4, STX > 4.

We tested the antimicrobial susceptibility of 146 *tet*(X4)-positive *E. coli* strains to 13 commonly used antibiotics. The MIC results showed that 145 *tet*(X4)-producing isolates were resistant to tigecycline and tetracycline. The MIC values for tigecycline range from 8 to 128 mg/L. Although carrying the *tet*(X4) gene, the strain 20PN649-3 was sensitive to tigecycline with a MIC of 0.5 mg/L. In addition, most of *tet*(X4)-positive *E. coli* were resistant to chloramphenicol (144/146, 98.63%), ampicillin (142/146, 97.26%) and trimethoprim/sulfamethoxazole (122/146, 83.56%). Resistance rates were lower for cefotaxime (45/146, 30.82%), ceftazidime (40/146, 27.40%), gentamicin (40/146, 27.40%), ciprofloxacin (28/146, 19.18%), fosfomycin (1/146, 0.68%) and imipenem (1/146, 0.68%). All *tet*(X4)-positive *E. coli* were susceptible to amikacin and colistin (Figure 1; Supplementary Table S1). These results indicated that these *tet*(X4)-positive *E. coli* were resistant not only to tetracycline antibiotics but also to a range of other antibiotics.

### Phylogenetic analysis of *tet*(X4)-positive *Escherichia coli* strains from various sources

**Figure 2:**
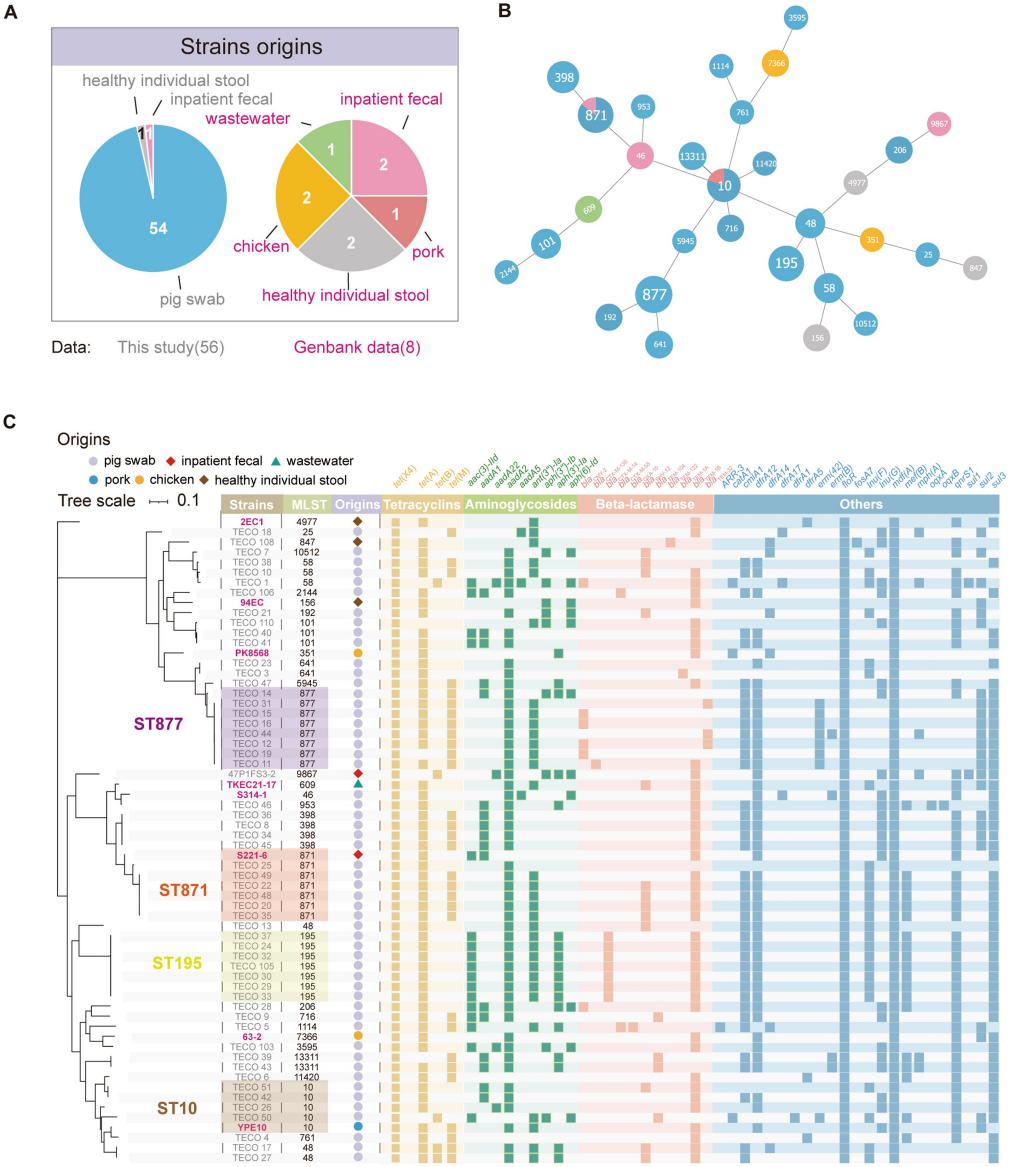
Population structure and resistance gene characteristics of 65 *tet*(X4)-positive *E. coli*. **(A)** Pie chart illustrating the distribution of sample sources. GenBank data accession numbers mentioned in this figure are listed in Supplementary Table S2A. **(B)** Minimum spanning tree based on MLST (Multilocus Sequence Typing). Each node in the tree represents a specific MLST type. The colors assigned to the nodes correspond to the strain’s origins, following the same color scheme as presented in **(A)**. **(C)** Phylogenetic tree and heatmap of resistance genes among the strains. The reference strains obtained from GenBank are highlighted in the tree.

Fifty-six *tet*(X4)-positive *E. coli* including 2 isolates from humans and 54 isolates randomly chosen from 144 *tet*(X4)-positive *E. coli* from pigs were further sequenced by whole genome sequencing. Among these, 54 strains were derived from two different farms (farm 1, *n* = 53 and farm 2, *n* = 1). To analyze the genomic differences between *tet*(X4)-positive *E. coli* strains from different sources, 8 *tet*(X4) positive *E. coli* strains of different sources obtained from GenBank were included in the dataset (Supplementary Table S2A). As mentioned above, 64 *tet*(X4)-positive *E. coli* strains used for phylogenetic analysis were sourced from healthy individual’s stool, inpatient fecal samples, pig swabs, chicken, pork, and wastewater (Figure 2A). The origins of these samples varied and clustered into 30 distinct STs (Figures 2B,C). In the strains collected in this study, the STs with the highest prevalence were ST877 (8/56, 14.29%), ST871 (6/56, 10.71%), ST195 (7/56, 12.50%), ST10 (4/56, 7.14%), and ST398 (4/56, 7.14%). The sample collected from inpatient belongs to ST9867. The ST871 strain S221-6 from inpatients in the public dataset showed close proximity in the phylogenetic tree to the corresponding ST871 strains collected in our study. Similarly, the ST10 strain YPE10 from pork in the public dataset exhibited a close relationship in the phylogenetic tree with the ST10 strains collected in our study. This finding indicated that *tet*(X4) had undergone extensive dissemination among multiple lineages of *E. coli*.

### Antimicrobial resistance gene (ARG) profiles of *tet*(X4)-positive *Escherichia coli*

A total of 60 resistance genes have been identified via whole genome sequencing. In the strains collected in this study, except for the *tet*(X4) gene found in all samples, most strains also carried *tet*(A) (49/56, 87.5%) (Figure 2C). Several resistance genes associated with aminoglycoside antibiotic resistance, including the widely carried *aad2* and *ant(3″)-la* genes, were identified. For beta-lactam antibiotics, a variety of resistance genes were detected, including *bla*_CMY-2_ (6/56, 10.71%), *bla*_CTX-M-14_ (7/56, 12.5%), *bla*_SHV-12_ (12/56, 21.43%), *bla*_TEM-1B_ (36/56, 64.29%). Furthermore, all strains harbored the florfenicol resistance gene *floR* and the macrolides resistance gene *mdf*(A). Additionally, high carriage rates were observed for the chloramphenicol resistance gene *cmlA1* (40/56, 71.43%), trimethoprim resistance gene *dfrA12* (41/56, 73.21%), fluoroquinolone resistance gene *qnrS1* (40/56, 71.43%), and sulphonamide resistance gene *sul3* (46/56, 82.14%) (Supplementary Table S3).

### Various genetic elements of *tet*(X4) in *Escherichia coli*

**Figure 3:**
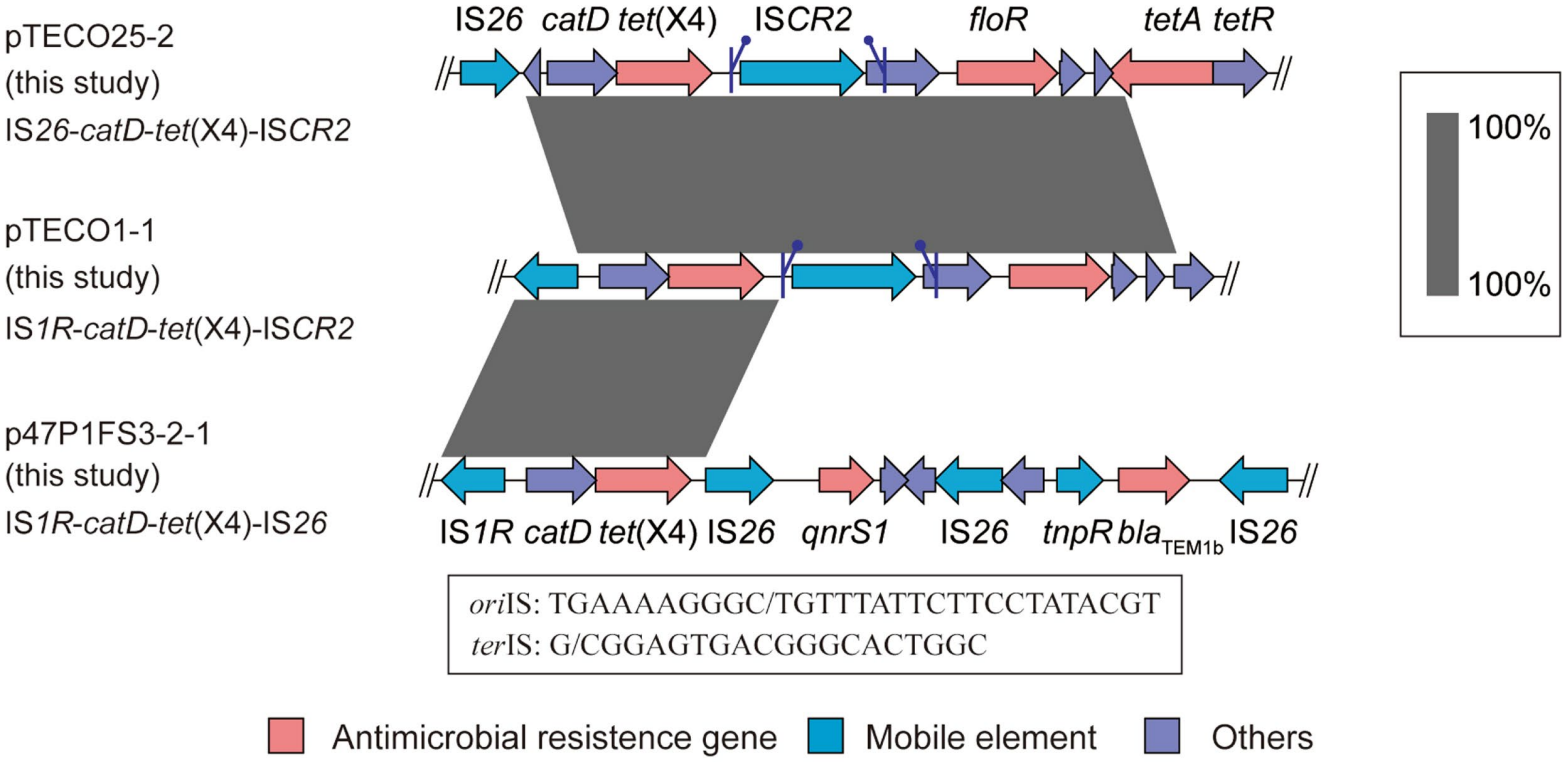
Comparison of genetic contexts of *tet*(X4). The schematic diagram provides a comparative analysis of genetic contexts surrounding *tet*(X4) in three strains subjected to short-read sequencing (TECO25, TECO1, and 47P1FS3-2). The symbols flanking the IS*CR2* represent the putative *ori*IS and *ter*IS of the IS*CR2, respectively.*

**Figure 4:**
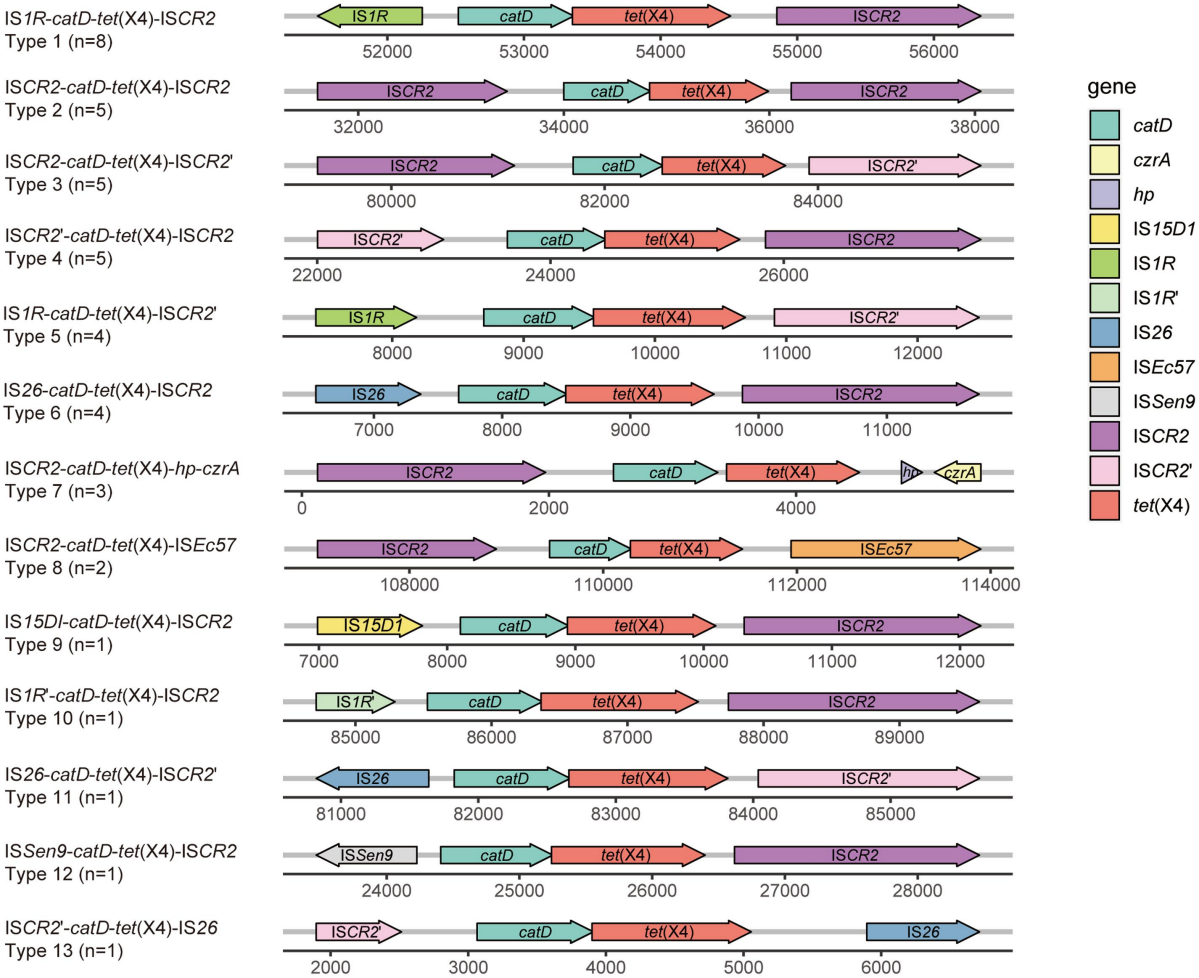
Different types of the genetic environments of *tet*(X4). The figure represents a comparison of the genetic environments of *tet*(X4) among 41 *tet*(X4)-positive *E. coli* obtained from GenBank. GenBank data accession numbers mentioned in this figure are listed in Supplementary Table S2C. The IS*1R*’ in this figure indicates that the IS*1R* gene fragment is incomplete, potentially due to truncation caused by certain insertion sequences. The representation method mentioned above is equally applicable to the incomplete genes present in this figure and Figure 5, including IS*CR2*’, IS*Pst2*’, *TnAs1*’, and *TnAs3*’.

The transfer of *tet*(X4) is frequently associated by surrounding mobile elements. To investigate the genomic region housing *tet*(X4) and to observe the associated mobile elements, we conducted a comparative analysis of the genetic environment surrounding *tet*(X4) in the 3 isolates investigated in this study. Notably, we identified distinct structures in the 3 *tet*(X4)-haboring plasmids including IS*26*-*catD*-*tet*(X4)-IS*CR2* (pTECO25-2), IS*1R*-*catD*-*tet*(X4)-IS*CR2* (pTECO1-1), and IS*1R*-*catD*-*tet*(X4)-IS*26* (p47P1FS3-2-1) (Figure 3). This finding revealed that IS*CR2*, IS*1R* and IS*26* combined into three different genetic contexts, indicating a diverse surrounding environment worthy of further exploration. Subsequently, following the published manuscripts from 2019 to 2022 related to *tet*(X4)-positive *E. coli*, we downloaded 398 *tet*(X4)-positive *E. coli* assembly data from GenBank, among which there were 41 samples with a single copy of *tet*(X4) and a complete surrounding genetic environment (Supplementary Tables S2B,C). Thirteen *tet*(X4)-harboring genetic contexts were identified (Figure 4). The major genetic contexts identified were IS*1R*-*catD*-*tet*(X4)-IS*CR2* (Type1) and IS*CR2*-*catD*-*tet*(X4)-IS*CR2* (Type2), which are consistent with the findings of other researchers (Liu et al., 2022). Mobile elements such as IS*1R*, IS*CR2*, IS*26* and their corresponding truncated elements are distributed around *tet*(X4), facilitating the propagation of *tet*(X4).

### Comparison of plasmids carrying *tet*(X4)

**Figure 5:**
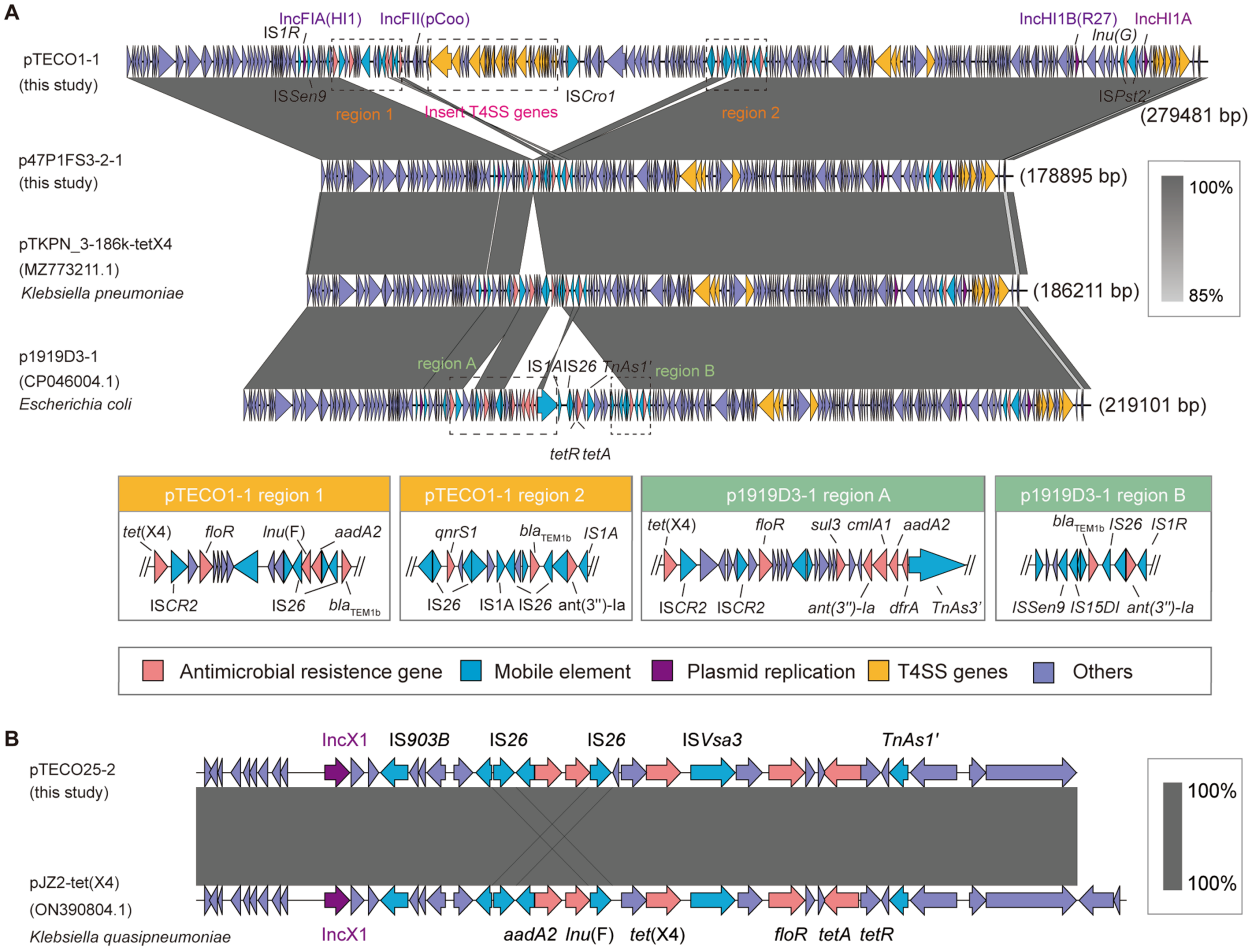
Structures of *tet*(X4)-positive plasmid. **(A)** The schematic diagram provides a comparative analysis of pTECO1-1, p47P1FS3-2-1, pTKPN_3-186k-tetX4 (MZ773211.1), and p1919D3-1 (CP046004.1). Different colored arrows representing different gene categories. Some regions where resistance gene insertions occur in pTECO1-1 and p1919D3-1 are indicated separately. **(B)** The schematic diagram provides a comparative analysis of pTECO25-1, pJZ2-*tet*(X4) (ON390804.1).

To provide a comprehensive view of the plasmids carrying *tet*(X4), we presented the complete plasmid structures containing *tet*(X4) from 3 strains (TECO25, TECO1, and 47P1FS3-2). These plasmids are pTECO1-1 (279,481 bp), p47P1FS3-2-1 (178,895 bp), and pTECO25-1 (29,625 bp). p47P1FS3-2-1, which had IncFIA(HI1)-IncHI1B(R27)-IncHI1A replicon type, carried the resistance genes *tet*(X4), *qnrS*, *bla*_TEM-1B_, *ant(3″)-la*, *lnu(G)*. pTECO1-1 (178,895 bp), had an IncFIA(HI1)-IncFII(pCoo)-IncHI1B(R27)-IncHI1A replicon type. Additionally, pTECO1-1 carried *floR*, *aac(3)-IId*, *lnu(F)*, *aadA2* genes, in addition to all the resistance genes on p47P1FS3-2-1. pTECO25-1, with replicon type IncX1, carried the resistance genes *aadA2*, *Inu(F)*, *tet*(X4), *floR*, *tet*(A) (Figure 5). The *bla*_CMY-2_ and *bla*_CTX-M-14_, mentioned earlier, were not located on these *tet*(X4)-positive plasmids.

BLAST analysis of p47P1FS3-2-1 against the NCBI database revealed that the plasmid p1919D3-1 (CP046004.1) had 98% coverage and 100% identity, whereas the plasmid pTKPN_3-186k-*tet*(X4) (MZ773211.1) had 100% coverage and identity. Their replicon types were all IncFIA(HI1)-IncHI1B(R27)-IncHI1A, suggesting that these strains shared a plasmid backbone with our strains. BLAST alignment results showed that these 4 plasmids were highly conserved at both ends, and had different fragment insertions downstream of *tet*(X4) mediated by variable insertion sequences, such as IS*26*, IS*1R*, IS*CR2*, and IS*15DI* (Figure 5A). This region was diverse, including a multidrug resistance region in p1919D3-1 and a region containing a plasmid replicon in pTECO1-1. Comparison of *tet*(X4)-positive plasmids showed a 100-kbp large fragment insertion at the back end of *tet*(X4), containing a replicon and a 40-kbp length of the T4SS transfer-related gene cluster (Supplementary Figure S1). *Klebsiella quasipneumoniae* pJZ2-*tet*(X4) (ON390804.1) was identified by performing BLAST of pTECO25-1 against the NCBI database, with 100% coverage, 99.8% identity (Figure 5B), indicating that cross-species transmission of this plasmid has occurred.

## Discussion

The environment serves as a vast reservoir for drug resistance genes and plays a crucial role in their transmission (Lin et al., 2021). Since its discovery, *tet*(X4) has been extensively detected in environment samples (Zhang et al., 2022). Regarding the transmission of *tet*(X4) from the environment to human, potential routes have been proposed in previous studies. These include: contamination of agricultural land or water sources such as reservoirs and wetlands through animal manure, subsequently leading to human exposure via crops or aquatic organisms, such as freshwater fish and shrimp (Li et al., 2019; Dao et al., 2022). Additionally, the presence of contaminated retail meat into the market had been identified as a potential transmission pathway (Sun et al., 2021). Direct contact or fecal-oral transmission among individuals involved in the food processing chain is also considered a possible route (Wang et al., 2020). Our study highlights the possibility of transmission from pork processing plants to workers, emphasizing the importance of implementing prevention and control measures among individuals working in relevant industries. The plasmids p1919D3-1 and pTKPN_3-186k-*tet*(X4) were derived from strains isolated from swab samples of pig feces from Henan province and pig nasal swabs from Guangdong province, China, respectively. The similarity of these plasmid backbones to the plasmid p47P1FS3-2-1 originating from the patient in our study suggests the potential transmission and evolution of *tet*(X4)-positive plasmids across different regions in China and between animals and humans.

*tet*(X4) is highly prevalent in China, with the detection of strains carrying *tet*(X4) including *E. coli*, *Klebsiella pneumoniae*, *Citrobacter braakii*, *Enterobacter cloacae* (Li et al., 2021; Wu et al., 2022). The prevalence of *tet*(X4)-positive *E. coli* in China exhibited significant diversity in STs with regional variations. In two provinces in northwestern China, Shanxi and Ningxia, the dominant STs of *tet*(X4)-positive *E. coli* were ST6704, ST2035, ST48, ST1602, and ST877 (Sun et al., 2020). In western China, ST10, ST34, ST48, and ST195 have been identified as predominant STs (Feng et al., 2022), while in the southern region, ST10, ST48, ST877, and ST2144 were relatively more prevalent (Cui et al., 2022). In our study, we isolated *tet*(X4)-positive *E. coli* strains from Guangdong Province in southern China, where we observed high proportions of ST877, ST871, ST195, and ST10. The diverse range of STs indicates that the horizontal transfer of plasmids carrying *tet*(X4) has likely occurred, contributing to the genetic variation and dissemination of this resistance gene. Moreover, the two *tet*(X4)-positive *E. coli* strains isolated from the patients in this study exhibited a unique ST (ST9867), which has not been previously reported, indicating the transfer of *tet*(X4) to a novel ST and its subsequent spread to human populations.

A transposable unit is formed when *tet*(X4) is transferred, and is generally composed of mobile elements, *tet*(X4) and partner gene *catD*, in which mobile elements are the key mediators of the transfer. Previous studies have demonstrated that IS*CR2*, through its upstream and downstream *ori*IS and *ter*IS elements, plays a crucial role in mediating the transfer of *tet*(X4) by forming a transposon unit that facilitates rolling-circle transposition (Toleman et al., 2006; Sun et al., 2020). Additionally, under the influence of other IS elements and homologous recombination events, IS*CR2* generates a highly diverse genomic environment. This includes the formation of tandem multicopies due to transposase misreading of *ter*IS, capture of other resistance genes, and truncation by other IS elements (Liu et al., 2022; Zhai et al., 2022). In this study, all the isolated strains carrying a single copy of *tet*(X4). Using the long-reads sequencing, we identified three transposon units comprising the different mobile elements. Combining our data with available online resources, we found that, in addition to IS*CR2*, IS*1R* and IS*26* elements were also frequently involved in mediating the transfer of *tet*(X4). Furthermore, we discovered that the presence of the florfenicol resistance *floR* gene frequently accompanied *tet*(X4) downstream, indicating that the dissemination of *tet*(X4) plasmids may be influenced by the selective pressure exerted by florfenicol.

One way in which antibiotic resistance plasmids acquire additional resistance genes is through the frequent insertion of genetic fragments. During horizontal gene transfer, antibiotic resistance plasmids can acquire additional resistance genes by inserting new fragments, such as transposons or other mobile elements. In this study, four plasmids shared the same plasmid backbone, IncFIA(HI1)-IncHI1B(R27)-IncHI1A. However, compared to plasmid p47P1FS3-2-1, the other three plasmids underwent insertion of resistance gene fragments, including *aadA2*, *qnrS1*, and *Inu(F)*. The insertion of diverse resistance genes provided the *tet*(X4)-positive plasmid with increased adaptability. In addition, the acquisition of an exogenous sequence containing a substantial number of T4SS-related genes by pTECO1-1 was a notable event. However, the significance of these findings requires further investigation.

This study has several limitations. First, the sample size was relatively small, as it included samples collected in Guangdong, China. Additionally, this study focused primarily on pigs, which lacked diversity in sample sources. This study also revealed the insertion of T4SS genes into a *tet*(X4)-positive plasmid, however, their functional significance requires further experimental verification.

## Conclusion

In this study, we described the high colonization rate of *tet*(X4)-positive *E. coli* in animals, along with its presence among the farm workers. Comparative analysis revealed that the successful genetic elements and the insertion of T4SS-associated genes in *tet*(X4)-positive plasmids, which may contribute to the rapid dissemination of *tet*(X4). Overall, this study serves as a timely warning regarding the potential acceleration of tigecycline resistance spread and underscores the critical importance of comprehensive surveillance spanning from animals to humans for effective global control of antibiotic resistance.

## Data availability statement

The sequences obtained in this study have been deposited in the GenBank database under BioProject number PRJNA1022166. Other public sequencing data used in this study are presented in Supplementary Table S2.

## Ethics statement

Ethical approval for this study was sought and given by both pig farm workers, inpatients and healthy volunteers. Individual consent forms were translated into Mandarin Chinese and the study vocally explained to each participant. All participants had the right to withdraw from the study at any stage.

## Author contributions

LS: Writing – review & editing, Methodology, Investigation, Writing – original draft, Conceptualization. CW: Writing – review & editing, Writing – original draft, Visualization, Software, Methodology, Conceptualization. CL: Writing – original draft, Methodology, Writing – review & editing, Validation, Formal analysis. RH: Writing – original draft, Investigation, Writing – review & editing, Validation, Formal analysis. GC: Writing – review & editing, Software, Methodology. DS: Writing – review & editing, Validation, Formal analysis. YY: Writing – review & editing, Software, Methodology. YF: Writing – review & editing, Validation, Formal analysis. GZ: Writing – review & editing, Software, Methodology. BY: Writing – review & editing, Validation, Formal analysis. MD: Resources, Writing – review & editing, Supervision, Project administration, Investigation, Conceptualization. G-BT: Resources, Writing – review & editing, Supervision, Project administration, Investigation, Conceptualization. L-LZ: Resources, Writing – review & editing, Supervision, Project administration, Investigation, Conceptualization.

## Footnotes

^1^ https://gitlab.com/antunderwood/contig_tools

^2^ https://github.com/tseemann/abricate

^3^ https://github.com/tseemann/mlst

^4^ https://isfinder.biotoul.fr/

^5^ https://tool-mml.sjtu.edu.cn/oriTfinder/

## Supplementary material

SUPPLEMENTARY FIGURE S1 Insertion Region of T4SS Genes in pTECO1-1. Insert T4SS genes region in Figure 5. The T4SS genes indicated in the figure are annotated by oriTfinder (https://tool-mml.sjtu.edu.cn/oriTfinder/oriTfinder.html).
