# Supplementary figures and images for: Genomic characterization revealing the high rate of tet(X4)-positive Escherichia coli in animals associated with successful genetic elements
Source: Front Microbiol. 2024 Jun 24;15:1423352. doi: 10.3389/fmicb.2024.1423352 (PMC11228144; doi:10.3389/fmicb.2024.1423352)

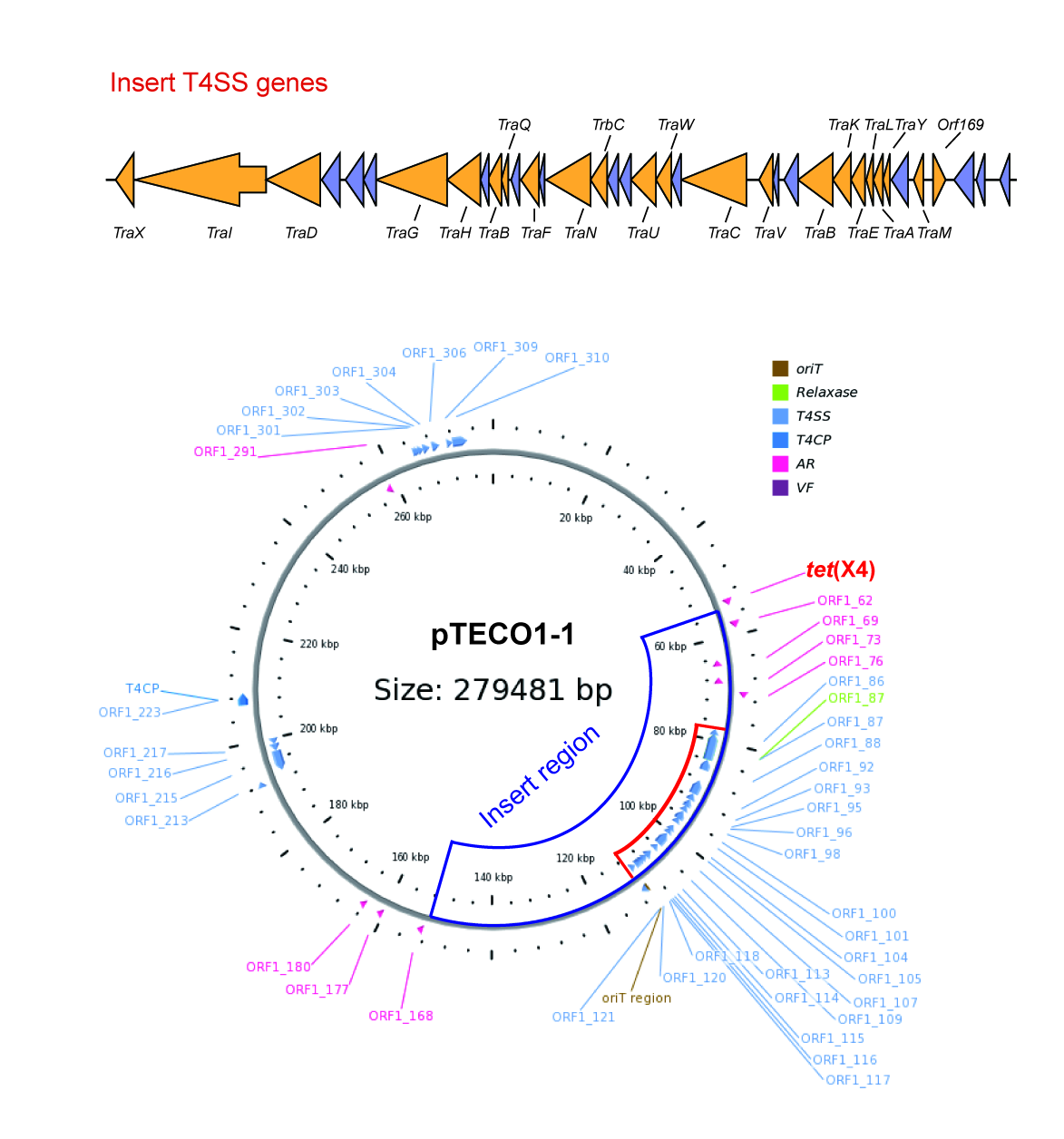

Supplement: SUPPLEMENTARY FIGURE S1 — Insertion Region of T4SS Genes in pTECO1-1. Insert T4SS genes region in Figure 5. The T4SS genes indicated in the figure are annotated by oriTfinder (https://tool-mml.sjtu.edu.cn/oriTfinder/oriTfinder.html). [file Image_1.TIF]
